# Differentially Expressed RNA from Public Microarray Data Identifies Serum Protein Biomarkers for Cross-Organ Transplant Rejection and Other Conditions

**DOI:** 10.1371/journal.pcbi.1000940

**Published:** 2010-09-23

**Authors:** Rong Chen, Tara K. Sigdel, Li Li, Neeraja Kambham, Joel T. Dudley, Szu-chuan Hsieh, R. Bryan Klassen, Amery Chen, Tuyen Caohuu, Alexander A. Morgan, Hannah A. Valantine, Kiran K. Khush, Minnie M. Sarwal, Atul J. Butte

**Affiliations:** 1 Department of Pediatrics, Stanford University School of Medicine, Stanford, California, United States of America; 2 Lucile Packard Children's Hospital, Palo Alto, California, United States of America; 3 Department of Pathology, Stanford University School of Medicine, Stanford, California, United States of America; 4 Division of Cardiovascular Medicine, Department of Medicine, Stanford University School of Medicine, Stanford, California, United States of America; Harvard Medical School, United States of America

## Abstract

Serum proteins are routinely used to diagnose diseases, but are hard to find due to low sensitivity in screening the serum proteome. Public repositories of microarray data, such as the Gene Expression Omnibus (GEO), contain RNA expression profiles for more than 16,000 biological conditions, covering more than 30% of United States mortality. We hypothesized that genes coding for serum- and urine-detectable proteins, and showing differential expression of RNA in disease-damaged tissues would make ideal diagnostic protein biomarkers for those diseases. We showed that predicted protein biomarkers are significantly enriched for known diagnostic protein biomarkers in 22 diseases, with enrichment significantly higher in diseases for which at least three datasets are available. We then used this strategy to search for new biomarkers indicating acute rejection (AR) across different types of transplanted solid organs. We integrated three biopsy-based microarray studies of AR from pediatric renal, adult renal and adult cardiac transplantation and identified 45 genes upregulated in all three. From this set, we chose 10 proteins for serum ELISA assays in 39 renal transplant patients, and discovered three that were significantly higher in AR. Interestingly, all three proteins were also significantly higher during AR in the 63 cardiac transplant recipients studied. Our best marker, serum PECAM1, identified renal AR with 89% sensitivity and 75% specificity, and also showed increased expression in AR by immunohistochemistry in renal, hepatic and cardiac transplant biopsies. Our results demonstrate that integrating gene expression microarray measurements from disease samples and even publicly-available data sets can be a powerful, fast, and cost-effective strategy for the discovery of new diagnostic serum protein biomarkers.

## Author Summary

Protein biomarkers in the blood are urgently needed for the diagnosis of a wide variety of diseases to improve health care. We aim to find a fast and cost-effective strategy to discover diagnostic protein biomarkers. Hundreds of diseases have already been investigated using microarray technology, measuring the mRNA expression of all genes in the disease-damaged tissues. We analyzed biopsy-based microarray data for 41 diseases in the public repository, identified genes with dysregulated mRNA expressions and detectable-protein abundance in the blood, and predicted them as candidate diagnostic protein biomarkers. We found that clinically and preclinically validated diagnostic protein biomarkers were significantly enriched in our predicted protein candidates for 22 diseases. We then measured the concentrations of ten predicted protein biomarkers in the serum samples from 39 renal transplant patients. Three of them were confirmed to be diagnostic of acute rejection after renal transplantation. All three proteins were further confirmed to be diagnostic of acute rejection in 63 cardiac transplant recipients. Our results show that publically available genome-wide gene expression data on disease-damaged tissues can be effectively translated into diagnostic protein biomarkers.

## Introduction

The utility of serum and plasma proteomic techniques to find diagnostic biomarkers has received considerable attention and investment in recent years. However, the limited sensitivity of mass spectrometers, the dynamic range of protein concentrations, and the presence of high abundance proteins in blood samples are major challenges in the identification and verification of potential protein biomarkers in peripheral blood [1].

Since the development of gene expression microarrays more than a decade ago [2], [3], many microarray studies have been used to study changes in mRNA transcripts in disease-related tissues. Considerable microarray data have been deposited into international repositories including the Gene Expression Omnibus (GEO) [4] and ArrayExpress [5], with at least 30% of US mortality already covered [6]. Integration of publicly-available microarray data has been used to show commonalities across cancers [7], suggest candidate gene variants associated with disease [8], associate relations with studied phenotypes [9], and even to validate gene-expression-based diagnostics for US Food and Drug Administration approval [10]. Although microarrays measure the relative abundance of mRNA transcripts, their translated proteins are also likely to be differentially present in diseased tissue and possibly even secreted or detectable in the blood. Rhodes et al first proposed to predict serum protein biomarkers by integrating cancer gene expression data from Oncomine and filtering the list with Gene Ontology annotation of “extracellular”, “extracellular matrix”, and “extracellular space” [11]. They predicted ten serum protein biomarkers for ovarian cancer and found that PRSS8 had previously been known to be an accurate biomarker for ovarian carcinoma.

We have previously developed a methodology to determine gene expression signatures across 238 diseases from GEO. We have found that the molecular signature of disease-specific RNA across tissues is more prominent than the signature of tissue-specific expression patterns [12]. We hypothesized that RNA measurements in diseased tissue could be used to identify candidate serum protein biomarkers of disease. We also hypothesized that integrating data sets from similar conditions could be used to find protein biomarkers applicable across the related conditions. Finally, we tested whether focusing only on those genes that code for proteins known to be detectable in serum and urine (here termed biofluids), using previously published resources might improve our specificity [13]. We then evaluated the general and specific performance of this Integrated RNA Data Driven Proteomics (IRDDP) method to suggest protein candidates across hundreds of diseases.

One field in urgent need of non- or minimally invasive protein biomarkers is solid-organ transplantation [14]. The diagnosis of acute allograft rejection (AR) is currently based on functional and histological grounds. The latter approach requires an invasive procedure in order to obtain sufficient representative tissue for pathology [15], [16], though blood-based RNA diagnostics are successfully being validated [17]. Additional serum biomarkers are still needed to reduce or avoid invasive diagnostic procedures for AR [16], [18], [19]. Many efforts have been made to identify such biomarkers [20]. For example, in renal transplant rejection, significantly increased protein concentrations of VEGF have been observed in serum and urine [21], [22], [23]. Increases in the following other entities have also been observed: CXCL9 in urine [24], soluble CD44 in plasma [25], ADL in serum [26], and TNF-alpha in serum [27]. HLA class I (ABC) protein levels were recently found to be elevated on the surfaces of peripheral blood CD3+/CD8+ T lymphocytes in AR at 14 and 21 days after renal transplantation [28]. However, there is still no reliable blood-based protein test to diagnose AR and none of these biomarkers has been shown to be universally present across all transplanted organs[14].

At the same time, a previous study also showed that there are similarities in the biology of the processes involved in the rejection of different transplanted solid organs [29]. Informed by these previous successes and efforts, we applied the IRDDP method here to search for blood-detectable proteins for acute rejection across different transplanted organs.

## Results

Our first goal was to test the hypothesis that blood- and urine-detectable protein biomarker candidates could be identified by using tissue-based gene expression microarray data. Using previously described methods [12], we acquired gene expression data sets representing 41 diseases, as well as control tissue samples for each from GEO [4], the largest international repository for gene expression microarray data with over 400,000 samples at the time of this writing.

We applied our IRDDP methodology to each disease. First, we calculated a set of differentially expressed genes for each disease using the RankProd meta-analysis package at a percentage of false prediction (pfp) ≤5% [30]. For diseases with multiple microarray data sets, we included genes that were differentially expressed in at least one of the data sets. We then filtered the gene sets through a list of 3,638 proteins with known detectable abundance in serum, plasma, or urine. The list was created from public sources [31], [32], [33], [34] and has been described [13]. This effort yielded a set of candidate protein biomarkers for each disease (Dataset S1).

**Figure 1:**
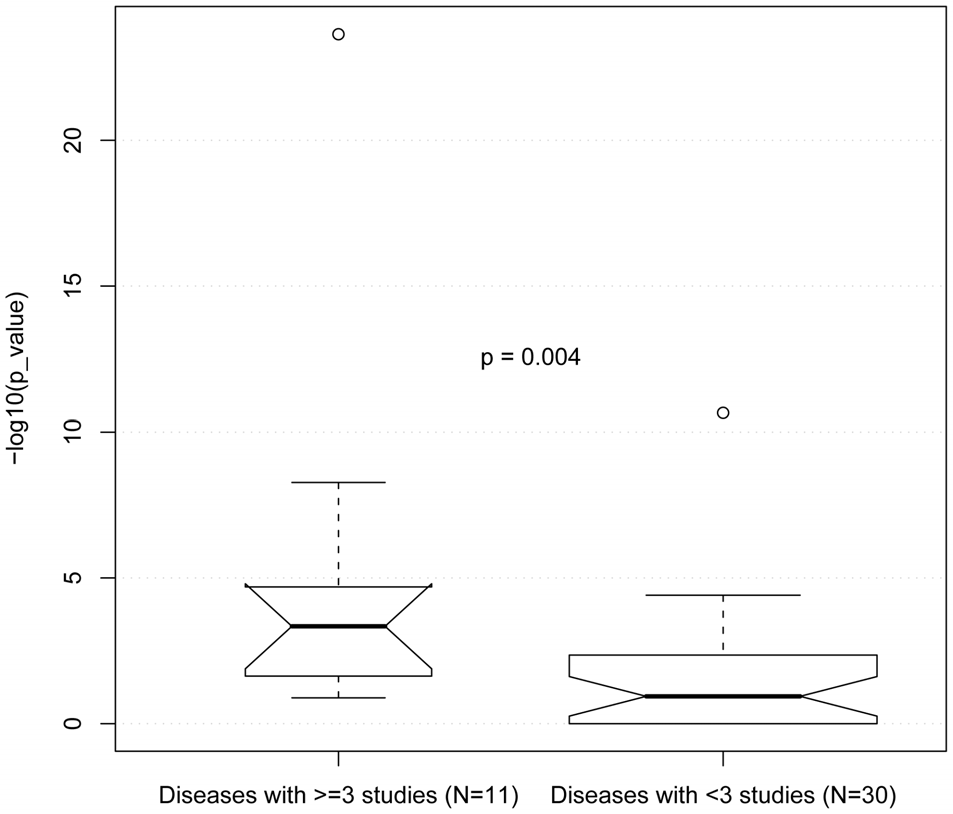
Identifying protein biomarkers was more likely if ≥3 gene expression data were available. We calculated Fisher's exact test association p-values between predicted and known protein biomarkers for each of 41 diseases (**Table 1**). The values are plotted on the y-axis. Known protein biomarkers were more likely to be rediscovered (Mann-Whitney U p-value = 0.004) for the 11 diseases represented by ≥3 gene expression data sets (left notched box plot, median p-value = 5×10^−4^), versus those 30 diseases represented by <3 data sets (right, mean p-value = 0.12). A notch was added around the median p-value in each box to indicate the significance of difference. When the notches about two medians do not overlap, the medians are roughly significantly different at about a 95% confidence level [54] . The circles are outliers.

For each disease, we then compared our candidate biomarkers with known diagnostic protein biomarkers in the GVK BIO Online Biomarker Database (GOBIOM). GIOBIOM is an independent manually curated knowledge base taken from global clinical trials, annual meetings, and journal articles [35]. As of this writing, GOBIOM contains 6,098 known biomarkers for 368 therapeutic indications with 23,166 unique references. For 22/41 diseases, known diagnostic protein biomarkers were enriched in our predicted protein sets (p<0.05, Fisher's exact, Table 1). In 9/11 diseases for which at least three data sets were available, known diagnostic protein biomarkers were even more significantly enriched in our predicted protein sets. The -log(p-value) in diseases with three or more data sets (n = 11) was significantly higher than those in diseases with fewer than three data sets (n = 30; p = 0.004, Fisher's exact, Fig. 1). Of the remaining 19 diseases, 11 were represented by only a single gene expression data set. Therefore, we concluded that the more gene expression datasets for a disease, the more likely known biofluid protein biomarkers are going to be significantly differentially expressed across any one of those data sets, suggesting the likelihood of finding new biomarkers increases with more available data sets. While this finding is not at all surprising, we were able to conclude that joining as few as three experiments could statistically significantly improve the performance to rediscover clinically validated protein biomarkers across 41 diseases.

**Table 1:** Known diagnostic protein biomarkers were significantly enriched in the sets of differentially expressed RNA for 22 out of 41 diseases.

Disease	GEO Accession Number	Predicted Protein Biomarkers*	Known Protein Biomarkers**	Overlap	P value$
Breast Cancer	GSE53, GSE1378, GSE1379, GSE1872, GSE2155, GSE2429, GSE2528, GSE3744, GSE4382	1845	134	63	2.26×10^−24^
Lung Cancer, Non-Small Cell	GSE1037	1064	44	23	2.21×10^−11^
Diabetes Mellitus, Type 2	GSE710, GSE642, GSE2470, GSE3068, GSE6428	439	17	9	5.30×10^−9^
Chronic Obstructive Pulmonary Disease	GSE475, GSE1650, GSE3320, GSE10964	217	18	5	5.14×10^−6^
Melanoma	GSE3189, GSE4587	1005	49	13	3.91×10^−5^
Alzheimer's Disease	GSE1297, GSE5281 (3 data sets)	1414	19	8	8.04×10^−5^
Crohn's Disease	GSE1710, GSE3365, GSE6731	1515	9	6	1.94×10^−4^
Cystic Fibrosis	GSE765, GSE769, GSE3100	234	8	3	4.56×10^−4^
Hypercholesterolemia	GSE3889	712	3	3	4.81×10^−4^
Wilm's tumor	GSE2712	192	2	2	6.53×10^−4^
Sickle Cell Anemia	GSE9877	1437	7	5	1.15×10^−3^
Myelodysplastic Syndromes	GSE2779, GSE4619	779	10	4	1.88×10^−3^
Leukemia, Chronic Lymphocytic	GSE2466	671	21	7	2.90×10^−3^
Lung Cancer, Small Cell	GSE1037	986	4	3	4.44×10^−3^
HIV Infection	GSE2171, GSE2504, GSE6740	367	24	4	7.34×10^−3^
Prostate Cancer	GSE1413, GSE3868	302	88	6	0.012
Diabetes Mellitus, Type 1	GSE710, GSE1623, GSE1659, GSE2254, GSE4616	214	9	2	0.018
Lymphoma	GSE60, GSE3211	28	27	2	0.023
Transitional Cell Carcinoma	GSE3167	899	3	2	0.025
Liver Cirrhosis	GSE1843, GSE6764	905	4	2	0.028
Ulcerative Colitis	GSE1710, GSE3365, GSE6731	1301	7	3	0.030
Heart Failure	GSE1988	96	2	1	0.044
Colon Cancer	GSE2178, GSE4107	451	17	2	0.096
Rheumatoid Arthritis	GSE1919, GSE2053, GSE3592	307	15	2	0.098
Cardiomyopathy	GSE1869, GSE5406	1172	5	2	0.11
Thyroid Cancer	GSE5364	933	6	2	0.12
Obesity	GSE474, GSE4692, GSE4697	161	10	1	0.13
Atherosclerosis	GSE363	25	21	1	0.15
Sarcoidosis	GSE1907	369	7	1	0.51
Hypertension	GSE1674	10	11	0	1
Vitamin B12 Deficiency	GSE2779	3	2	0	1
Testicular Cancer	GSE1818	100	14	0	1
Bipolar Disorder	GSE5389	267	1	0	1
Schizophrenia	GSE4036	116	5	0	1
Leukemia, Acute Myeloid	GSE2191	148	11	0	1
Parkinson's Disease	GSE7621	100	6	0	1
Thymic Carcinoma	GSE2501	65	6	0	1
Obstructive Sleep Apnea	GSE1873	34	3	0	1
Osteoarthritis	GSE1919	60	3	0	1
Inflammatory Bowel Disease	GSE4183	619	2	0	1
Multiple Sclerosis	GSE10064	11	3	0	1

*Number of genes that were differentially expressed in any one of the disease tissues at the mRNA level (fpf≤0.05, RankProd R package) with detectable protein abundance in the biofluid proteome database (see Methods).

*Number of known diagnostic protein biomarkers in clinical and preclinical studies from the GVK BIO Online Biomarker Database (GOBIOM).

**Number of correctly predicted diagnostic protein biomarkers.

$ P values were calculated to evaluate whether known protein biomarkers were significantly enriched in our predicted genes using Fisher's exact test.

**Figure 2:**
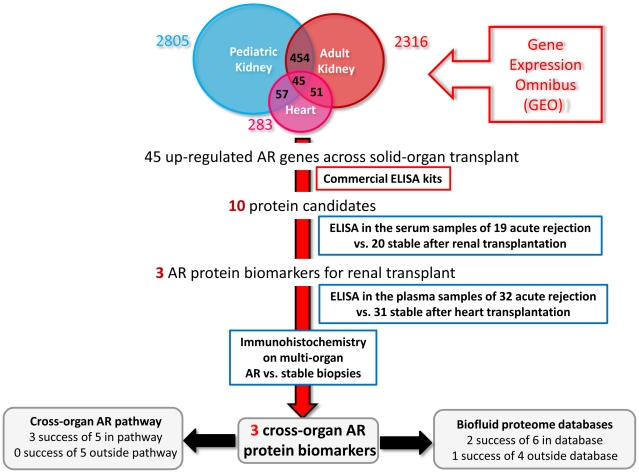
Identification of cross-organ AR protein biomarkers through integration of gene expression data. We integrated three microarray studies examining gene expression after rejection in the biopsy samples from pediatric renal, adult renal, and adult heart transplants (the latter two were retrieved from GEO). We identified 45 genes that were upregulated in common in acute rejection compared to stable graft function. Among ten proteins we tested by ELISA, the concentrations of three were higher in serum samples from AR patients. The concentrations of the same three proteins were also higher in AR samples from cardiac transplantation. Immunohistochemistry showed that PECAM1 was increased in AR vs. stable biopsies in renal, hepatic and cardiac transplantation. All three biomarkers were from our identified AR pathway, and two of them showed detectable protein abundance in the biofluid proteome database we constructed before. CXCL9 was not listed in our biofluid proteome database, but is known to have detectable protein abundance [24].

We then applied IRDDP to the specific problem of finding serum biomarkers for the diagnosis of transplant acute rejection (AR). We integrated three biopsy-based gene expression microarray studies from pediatric renal, adult renal [36], and adult cardiac [29] transplantation, identified genes commonly upregulated in AR compared to stable graft function, and then measured the abundance of proteins encoded by these genes in serum to identify cross-organ AR protein biomarkers (Fig. 2). The first of the three studies was performed in pediatric renal transplantation. It compared gene expression profiles in biopsy samples from 18 AR patients and 18 patients with stable graft function (STA) at the absence of AR and any other substantive pathology (Table S1). Using Significance Analysis of Microarrays [37], we found 2,805 genes with increased expression in AR biopsies (q-value ≤0.05; fold change ≥2).

We combined the results of this study with data from two other transplant studies that we retrieved from GEO. One study compared biopsy samples from 13 AR patients with 19 STA samples after adult kidney transplant (GEO dataset GDS724 [36]). The study yielded 2,316 upregulated AR genes with q-values ≤0.05. The second study compared 12 AR biopsy samples with 13 non-rejection samples after cardiac transplant (GEO series GSE4470 [29]). It yielded 283 upregulated AR genes with q-values ≤0.05. By intersecting the three data sets, we identified a gene expression signature containing 45 genes in common, irrespective of the specific studies or transplanted organs (Table S2). These genes are hereafter referred to as the “common-AR” set of genes.

To evaluate the significance of finding 45 genes in common, we shuffled the gene labels across the three data sets and repeated the entire analysis 100,000 times. In random performance, the number of intersecting genes was normally distributed around n = 9 (Fig. S1), suggesting a false discovery rate of 20%. This result also suggested that the probability of finding 17 or more commonly dysregulated AR genes by chance was less than 1%, and that the probability of finding 24 or more of them by chance was less than 1×10^−5^.

We next retrieved mRNA expression data for each common-AR gene across 74 tissue and cell types from SymAtlas [38], and identified the cell type with the highest expression. Surprisingly, our common-AR genes were most enriched in CD14+ monocytes (p = 0.003, Fisher's exact). Seven of the 45 common-AR genes had their highest expression levels in CD14+ monocytes: they were *CD44*, *IL10RA*, *S100A4*, *IGSF6*, *CTSS*, *CASP4*, and *SCAND2*. Our results suggest an important role for activated pro-inflammatory monocytes in transplant rejection. This finding is consistent with recent reports that monocyte/macrophage activation might induce inflammation, leading to impairment of graft function in renal transplant patients [39].

We then analyzed the functions of the 45 common-AR genes using Ingenuity Pathway Analysis. As expected, 28 of the 45 common-AR genes were involved in the inflammatory response (p = 3.37×10^−17^, Fisher's exact; p<3.56×10^−3^ after Benjamini-Hochberg multi-test correction). Furthermore, 23 common-AR genes were involved in cell-mediated immune responses, (p = 3.34×10^−15^; p<2.97×10^−3^, Benjamini-Hochberg correction). Finally, 23 common-AR genes were involved in a single pathway associated with inflammatory responses, antimicrobial responses, and cellular movement regulated by STAT-1 (Fig. S2).

ELISA kits were available for ten of the 45 candidate proteins, including six proteins known to be in biofluids and four outside. We measured all ten proteins in a pilot study of serum samples collected within 24 hours after biopsy from an independent set of 19 patients with biopsy-proven AR and 20 patients with absence of AR or any other substantive pathology (STA). The patients were from a pediatric and young adult renal transplant study. No patients were positive for BK virus infection, and no patient samples in the ELISA study were matched with samples used in the microarray study. The AR/STA samples were matched for recipient and donor gender, age, type of immunosuppression, time post-transplant, race, and type of end stage renal disease (Table S3).

**Figure 3:**
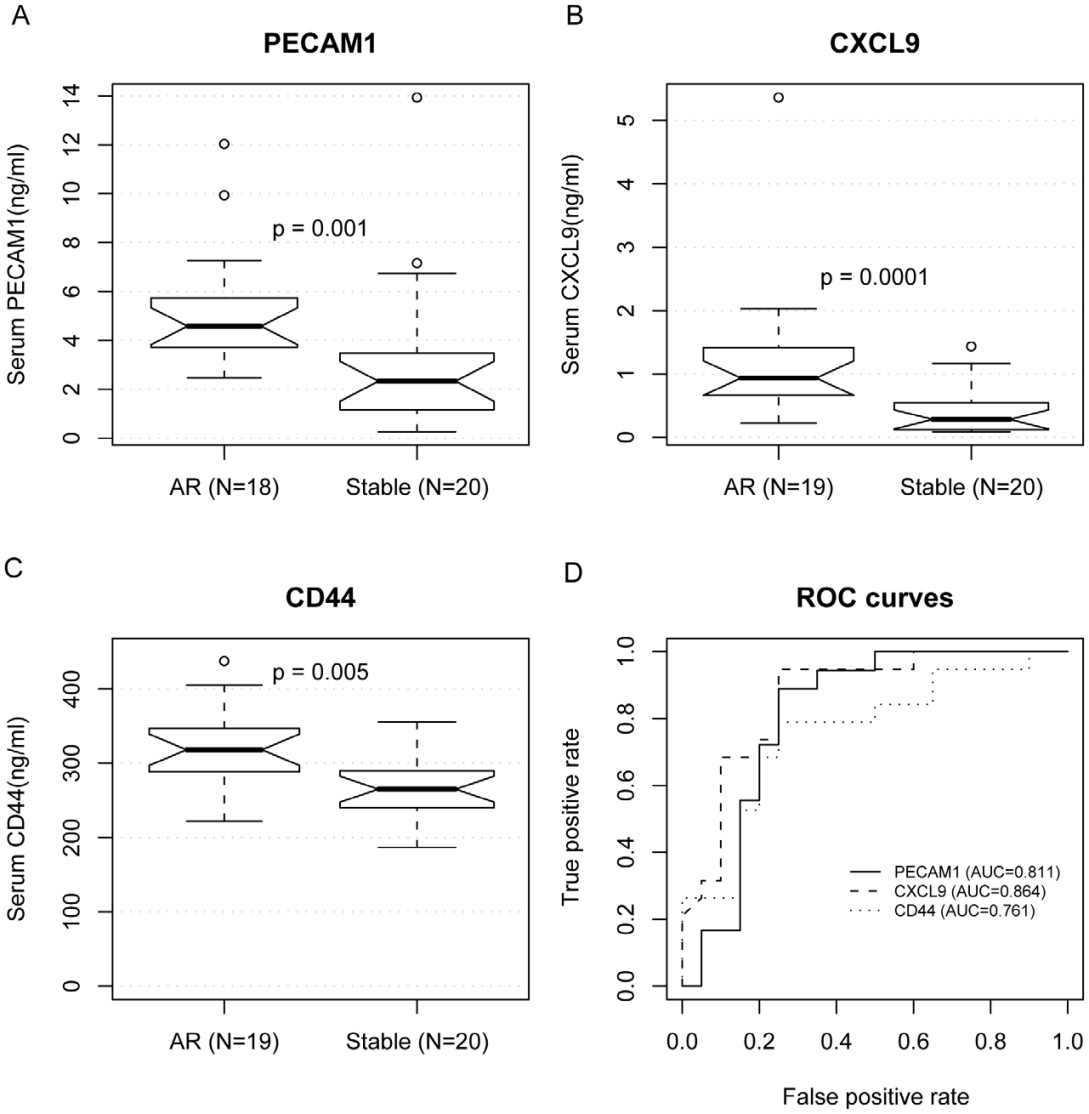
Serum ELISA results of three protein biomarkers in renal transplantation. We measured the protein concentration of ten genes by ELISA in independent serum samples of 19 AR patients and 20 patients with stable (STA) graft function after renal transplant. The protein concentrations of PECAM1 (A), CXCL9 (B), and CD44 (C) were higher in the AR serum samples, as shown in the notched boxplots. When the notches about two medians do not overlap, the medians are roughly significantly different at about a 95% confidence level [54] . The circles are outliers. P-values were calculated using the Mann-Whitney U test. One of the AR samples was inadvertently lost during the PECAM1 experiment and could not be recovered. (D) Areas under the Receiver Operating Characteristic (ROC) curves used to distinguish AR from STA were 0.811, 0.864, and 0.761 for PECAM1, CXCL9, and CD44, respectively.

Three of the ten proteins were statistically significantly upregulated in the AR serum samples compared to the STA samples after renal transplantation (Fig. 3). They were PECAM1 (also known as CD31 antigen, or platelet/endothelial cell adhesion molecule), CXCL9 (MIG, chemokine ligand 9), and CD44 (hyaluronic acid receptor). Mann-Whiney U test for significant differences yielded p-values of 1×10^−3^, 1×10^−4^, and 5×10^−3^, respectively. Receiver Operating Characteristics (ROC) curves showed the ability of each individual protein to distinguish AR from STA (Fig. 3d). The areas under the ROC curves (AUC) were 0.811, 0.864, and 0.761 for PECAM1, CXCL9, and CD44, respectively. At optimal performance, PECAM1 distinguished AR from STA with 89% sensitivity and 75% specificity; CXCL9: 78% sensitivity and 80% specificity; CD44: 80% sensitivity and 75% specificity.

**Figure 4:**
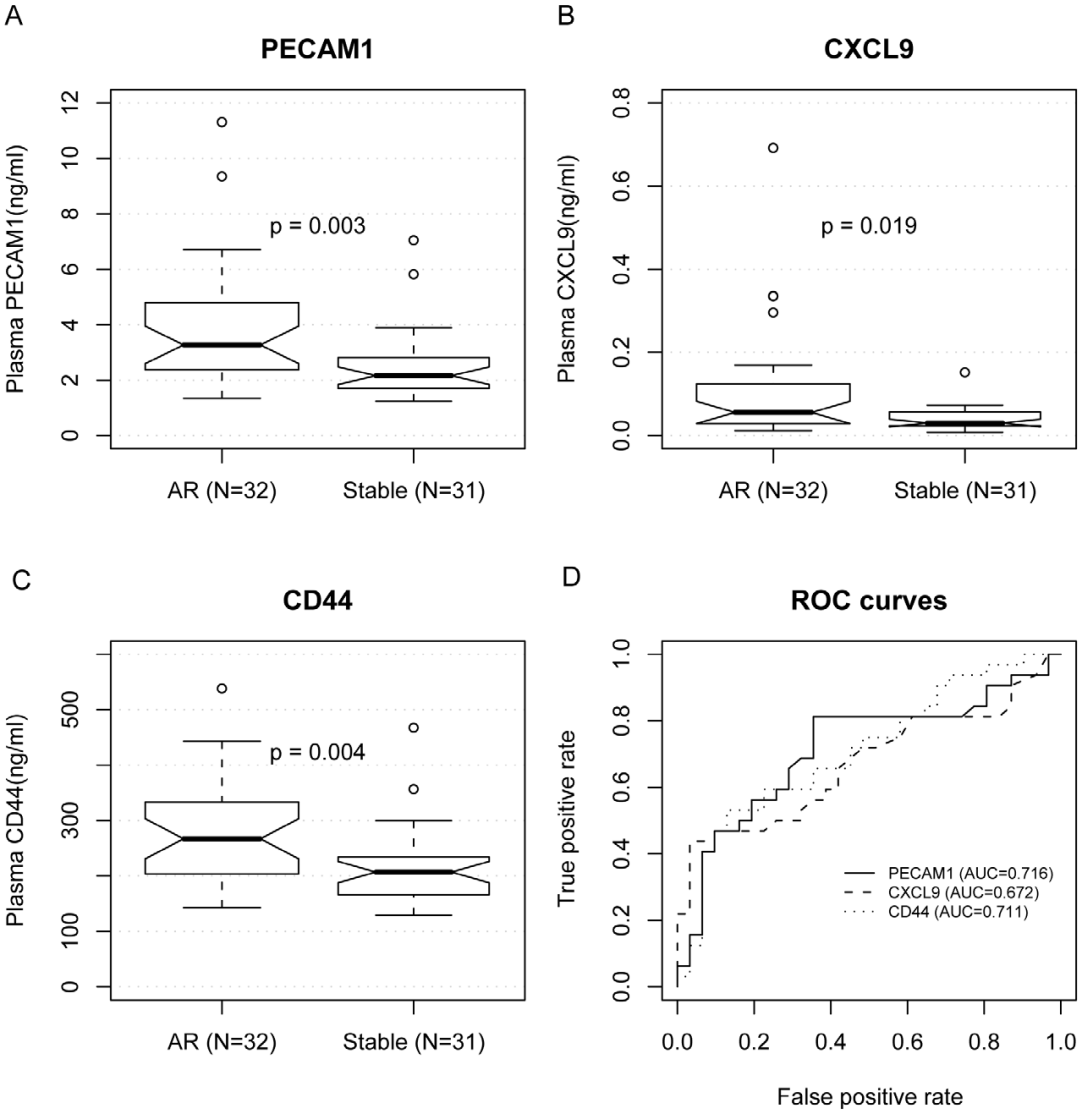
Plasma ELISA of three protein biomarkers in cardiac transplantation. We measured the protein concentrations of PECAM1 (A), CXCL9 (B) and CD44 (C) by ELISA in the plasma of 32 AR patients and 31 STA patients after cardiac transplantation. All three proteins have statistically significantly higher concentration in the AR serum samples, compared to STA as shown in the notched box plots. P-values were calculated using the Mann-Whitney U test. (D) In ROC curves used to distinguish AR from STA, the areas under the curves were 0.716, 0.672, and 0.711 for PECAM1, CXCL, and CD44, respectively.

We then measured the concentration of these proteins in a second pilot study on plasma samples of cardiac allograft recipients to identify cross-organ AR biomarkers. We compared samples from 32 AR patients and 31 STA patients. The samples were matched for demographic characteristics (Table S4). None of them was infected with CMV. Interestingly, all three markers were upregulated in AR compared to STA. Mann-Whitney U test for significant differences yielded p values of 3×10^−3^ (PECAM1), 0.019 (CXCL9), and 4×10^−3^ (CD44) (Fig. 4). The areas under the ROC curves for distinguishing AR from STA were 0.716, 0.672, and 0.711 for PECAM1, CXCL9, and CD44, respectively.

We evaluated the performance of a combined panel of PECAM1 and CXCL9 using a three-fold cross-validation. We randomly selected two thirds of the samples, trained a multinomial logistic regression model, and calculated the predictive performance on the remaining one third of samples. After repeating the process 1000 times, the average ROC curves showed an improvement on cardiac AR diagnosis and no additional improvement on renal AR diagnosis (Fig. S3), suggesting a large clinical trial combining PECAM1 and CXCL9 with other previously found protein biomarkers would be needed to evaluate the predictive diagnosis of AR. Adding CD44 did not improve the regression models.

**Figure 5:**
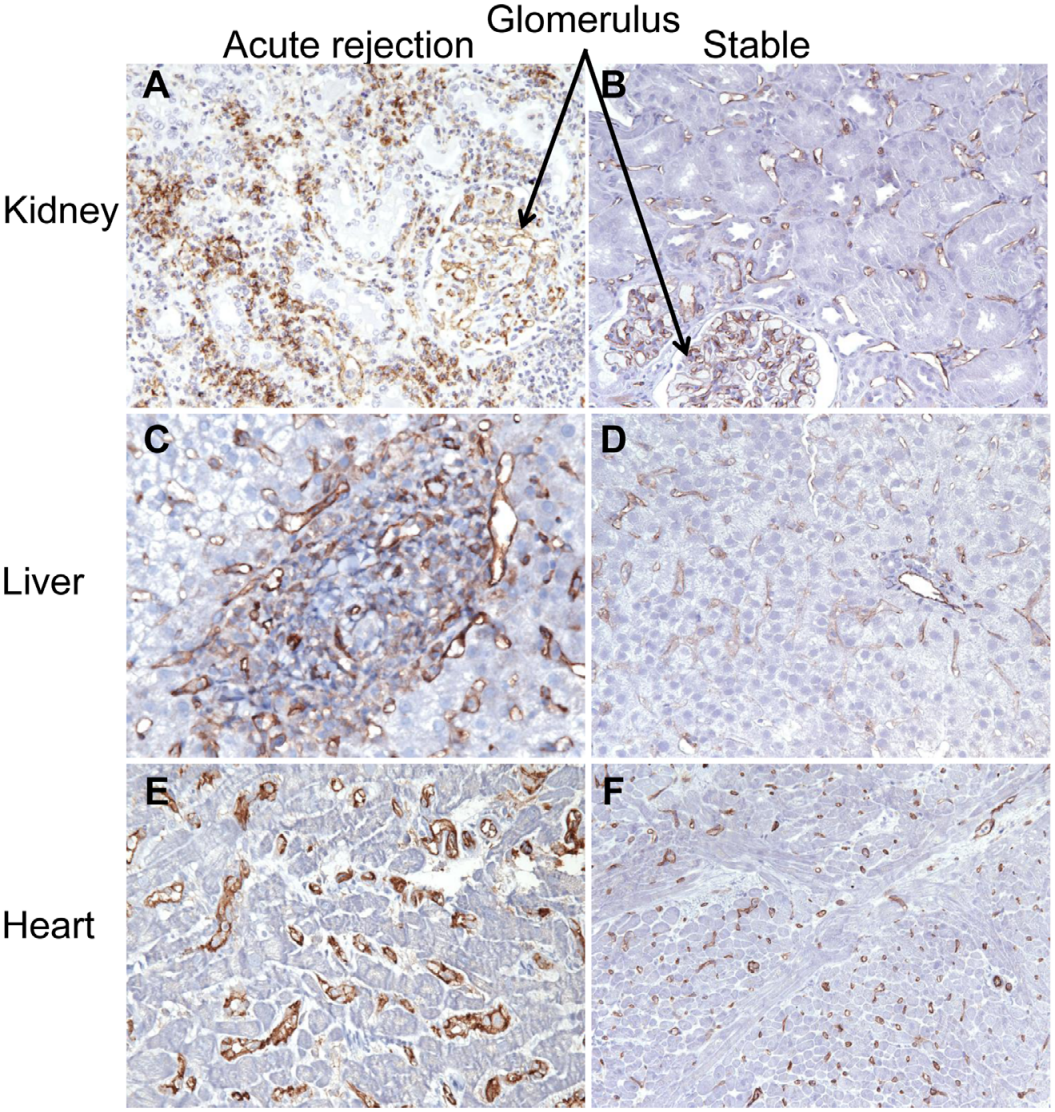
*In situ* PECAM1 staining in acute rejection and stable patients in renal, hepatic, and cardiac allograft biopsies. (A) Acute rejection in a renal allograft biopsy with PECAM1 positive infiltrating lymphocytes and monocytes; endothelial cell staining occurred in glomeruli and peritubular capillaries. (B) In a stable graft renal allograft biopsy, PECAM1 staining occurred only in endothelial cells in glomeruli and peritubular capillaries. (C, E) Dense staining was observed in AR tissues after hepatic (C) and cardiac (E) transplants in infiltrating mononuclear cells and endothelial cells of capillaries and larger blood vessels. In hepatic (D) and cardiac (F) transplant biopsies from stable grafts, weak endothelial cell staining was observed (magnification ×400).

We performed an immunohistochemistry study on our best-performing marker, PECAM1. The goal of the study was to compare its protein expression in AR and STA samples from renal, hepatic and cardiac allograft biopsies (Fig. 5). In STA kidney tissue, PECAM1 staining was mainly observed in the endothelial cells of glomeruli, in peritubular capillaries, and in large blood vessels. In contrast, examination of staining patterns in AR biopsies revealed dense infiltrates of PECAM1, as well as positive lymphocytes and mononuclear cells in the interstitium. Similarly, dense endothelial PECAM1 staining was observed in the hepatic and cardiac transplant AR tissues, along with staining in infiltrating mononuclear cells. We observed only minor endothelial staining in hepatic and cardiac STA tissues. These immunohistochemistry results showed significantly increased PECAM1 protein expression in the AR tissues compared to STA tissues across transplanted organs.

Furthermore, our studies showed that PECAM1 protein was also significantly upregulated in the serum samples from AR patients compared with samples from patients with BK virus infection (n = 10, p = 0.001, Mann-Whitney U test) and chronic allograft injury (n = 10, p = 6×10^−5^, Mann-Whitney U test) after renal transplantation (Fig. S4). Analysis across hundreds of diseases using our GeneChaser tool [40] showed that the mRNA expression of *PECAM1* is significantly upregulated in various cancers, but not in other potential confounding conditions, such as infection and hypertension (http://tinyurl.com/yhq9h3k). These results suggest that PECAM1 is a serum marker specific for allograft acute rejection, irrespective of the transplanted organ.

Finally, as mentioned above, 23 of our 45 common-AR genes were involved in a single pro-inflammatory pathway regulated by STAT-1 (Fig. S2). Among the ten proteins we tested by ELISA, five were within this pathway and five were outside of it. All five proteins outside the pathway failed validation, while three of the five proteins inside it were validated as AR markers. The 60% success rate from within this single pathway suggests that it is likely to represent a common functional pathway in AR across transplanted organs. Other novel AR protein markers are likely to be found from the remaining 18 common-AR genes/proteins inside this pathway that have not yet been tested by ELISA (Fig. S2). These proteins include CD2, Cathepsin S, and SH2D2A.

## Discussion

We developed an Integrated RNA Data Driven Proteomics (IRDDP) method, which exploits the link between RNA changes in disease-affected tissue with serum detectable proteins coded by those RNA, yielding candidate proteins diagnostic for those diseases. We have demonstrated that this approach could be used to suggest candidate protein biomarkers for 22 diseases, and have shown the enrichment of known clinically and pre-clinically validated protein biomarkers in these candidate biomarkers. We applied our method to new and publicly-available microarray measurements on solid-organ transplantation, and identified and validated three cross-organ serum protein biomarkers for transplant rejection. Our results demonstrate that the integration of gene expression microarray measurements from disease samples, and even publicly-available data sets, can be a powerful, fast, and cost-effective strategy for discovering diagnostic serum protein biomarkers.

We found that PECAM1, CXCL9 and CD44 proteins were significantly upregulated in the serum/plasma samples of both renal and heart transplant patients with acute rejection compared with patients with stable graft function. The abundance of CXCL9 in urine [24] and that of soluble CD44 in plasma [25] have previously been shown to increase in renal AR compared with STA. In addition, macrophage surface PECAM1 can distinguish lung transplant rejection [41] but is not diagnostic in mouse models of cardiac transplant rejection [42]. But to our knowledge, this study is the first to show all three markers as cross-organ AR protein biomarkers in human serum or plasma. Our best marker, serum PECAM1, identified renal AR with 89% sensitivity, 75% specificity, 26% PPV, and 99% NPV at 9% prevalence[43], suggesting its potential clinical usage to monitor transplant patients to decrease the number of biopsies. We have focused on the biomarkers that were upregulated in AR because that was what most clinic tests are using. Proteins downregulated in AR could potentially be used for diagnosis as well, and we have predicted both up and downregulated proteins for 44 diseases in Table 1 and Dataset S1.

We found that the likelihood of finding protein biomarkers indicative for a disease increases with the number of available gene expression datasets. Many meta-analysis methods have been shown to improve the identification of differentially expressed genes [44]. The identification of protein biomarkers might be improved through more sophisticated meta-analysis methods, such as the Rank Product method [30], measurements of concordance among data sets [45], and the identification of common features for diagnostic protein biomarkers. Filtering differentially expressed genes through known proteins detectable in biofluids may also improve specificity.

Future work will involve taking markers validated in our pilot studies of cross-organ AR and testing their clinical utility in blinded prospective studies. These studies might also elucidate the prognostic value of these markers. Given that hundreds of thousands of microarray measurements are now publicly available and that this number is growing, RNA data-driven proteomics could provide hundreds of serum and urine biomarkers for other diseases.

## Methods

### Ethics Statement

This study was approved by the Stanford University Institutional Review Board. Written informed consent was obtained from all the subjects.

### Identification of differentially expressed genes

As previously described [12], we identified microarray experiments containing both disease and normal control tissues for 280 diseases from the NCBI Gene Expression Omnibus (GEO) [4], calculated differentially expressed probes with percentage of false prediction (pfp) ≤5% using the RankProd R package [30], and converted probe IDs to Entrez Gene IDs using AILUN [46]. For genes with multiple probes, the probes with the most significant pfp values were used. For diseases with multiple data sets, we used genes that were differentially expressed in at least one data set.

### Prediction of protein biomarkers

We have previously constructed a human biofluid proteome database [13] with known serum- and urine- detectable proteins containing data from the HUPO Plasma Proteome Project [31], a non-redundant list from the Plasma Proteome Institute [32], the MAPU Proteome database [47], and the Urinary Exosome database [48]. We filtered the differentially expressed gene sets with our human biofluid proteome database to yield potential protein biomarkers for each disease.

### Enrichment of known protein biomarkers

We downloaded all diagnostic protein biomarkers from the GVK BIO Online Biomarker Database (GOBIOM) [35] with a selection of *Biochemical* in **Nature** and *Diagnosis* in **Application**, and limited the retrieval to those with valid Entrez Gene IDs annotated as *Protein* in **Chemical Nature**. We mapped clinical indications in this database to disease concepts represented in the Unified Medical Language System (UMLS) [49], and matched them to the disease concepts curated in our microarray data. We used the microarray data to identify 41 diseases with predicted biomarkers and are known protein biomarkers in GOBIOM. For each disease, we calculated the enrichment p-values between the predicted and known protein biomarkers using Fisher's exact test in R.

### Patients and samples

We collected 18 acute rejection (AR) and 18 stable (STA) biopsy samples from pediatric renal allograft recipients at the Stanford Hospitals, and measured gene expression profiles by microarrays. AR and STA samples were matched for recipient and donor gender, age, donor source, race, time post-transplant, HLA matches. Furthermore, all patients were under the same double (Tacrolimus and MMF) or triple immunosuppression protocols (Tacrolimus, MMF and steroid), and all had received Daclizumab induction therapy [50]. Mean and standard deviation data for patient demographic and clinical variables are provided in Table S1. The difference in the sample collection time between AR and STA was caused by two AR samples collected at 69 and 97 months after transplant. The remaining 16 AR samples were collected at 9±7 months after transplant, the same as that of stable patients. Removing the two late-stage AR samples only caused minor changes in the AR signature. A sample was categorized as AR with biopsy proven according to the Banff classification [51] on tubulitis, interstitial inflammation, glomerulitis, and vasculitis (n = 18, Banff grade samples were IA, IB, and IIA not including border line). Samples were categorized as STA (n = 18) if AR and any other substantive pathologies were absent. We also required stable graft function on protocol biopsy, which we conducted at 3, 6, 12, and 24 months after transplantation and for graft dysfunction [50], [52]. None of patients was infected with BK virus. All pathology analyses were performed by a single blinded pathologist (NK) at Stanford University.

For ELISA experiments on renal transplant serum samples, we used previously collected serum samples from 19 AR and 20 STA patients who were not infected with BK virus. All serum samples were obtained within 24 hours of a clinically indicated or protocol graft biopsy, and each sample was matched with the patient's biopsy. AR samples were biopsy-proven according to the Banff classification (IA, IB, IIA, IIB, not including border line). For specificity testing, an additional 10 samples were collected from patients with chronic allograft injury, who were defined as having an IFTA score ≥1 [51]. Ten samples were collected from renal transplant patients with BK virus infection. We also collected plasma samples from 32 AR patients and 31 STA patients without CMV infection after cardiac transplant at Stanford Hospitals. To minimize loss in sample processing, plasma was directly used in the ELISA study. Acquisition of samples in both studies was approved by the Stanford University Institutional Review Board. All AR samples were graded as ISHLT grade 3A or 3B. Stable samples showed an absence of AR and any other substantive pathology.

### Microarray experiments

Total RNA used for first-strand cDNA synthesis using a T7 promoter-linked oligo(dT) primer following the standard protocol for the Affymetrix One-Cycle cDNA Synthesis Kit (Affymetrix, Part. 900493). After second strand cDNA synthesis, biotin-labeled cRNA was prepared in an *in vitro* transcription reaction using a GeneChip IVT Labeling Kit (Affymetrix). Ten micrograms of fragmented cRNA was used for hybridization on Affymetrix Human Genome U133 Plus 2.0 microarrays according to the manufacturer's instructions. The raw and processed data have been deposited into GEO (accession ID; GSE14328).

### Microarray data analysis

All three datasets (pediatric renal, adult renal, and adult heart) were normalized by the quantile-quantile method using dChip software[53]. Probes significantly upregulated in AR versus STA were identified using Significant Analysis of Microarray (SAM; q ≤0.05) [37]. All probes associated with AR were linked to Entrez Gene IDs using AILUN [46]. We limited AR genes as significantly upregulated in AR compared to STA. We found 9,086 genes associated with pediatric renal AR, 2316 in adult renal AR, and 283 in heart AR.

The number of heart AR genes was significantly less than those of kidney AR genes due to different platforms and organs. Publicly available heart AR data came from studies that used a 70mer spotted array from NIH/NIAID (GEO accession numbers GPL1053 and GSE4470). The array contained 8972 probes that corresponded to 8437 Entrez Gene Ids. This array was smaller than the Affymetrix U133 plus 2.0 array used for the pediatric renal study and the Affymetrix U95 array used for the publicly available adult renal study (GEO accession numbers GPL91 & GDS724).

To make the number of AR genes comparable between pediatric and adult renal studies, we added an extra filter. We included only genes with a fold change ≥2 in the pediatric renal study. We obtained 2,805 pediatric renal AR genes, 2,316 adult renal AR genes, and 283 heart AR genes (Fig. 2). When we intersected these three AR gene lists, we found 45 common upregulated AR genes irrespective of transplanted organs.

### ELISA validation

Ten proteins in serum were measured by using commercial ELISA kits. ELISA kits for PECAM1 (Cat. No. ab45910), CD44 (Cat. No. ab45912), and SELL (Cat. No. ab45917) were purchased from ABCam Inc (Cambridge, MA); an ELISA kit for SA100A4 (Cat. No. CY-8059) was purchased from MBL International (Woburn, MA); ELISA kits for CCL4 (Cat. No. DMB00), CXCL11 (cat. No. DCX110) and CXCL9 (cat. No. DCX900) were purchased from R&D Systems (Minneapolis, MN). An ELISA kit for STAT-1 (cat. CBA034) was purchased from Calbiochem (Gibbstown, NJ); an ELISA Kit for BIRC5/Survivin (Cat. No. 900-111) was purchased from Assay Designs (Ann Arbor, MI), and an ELISA assay for CCL8 was developed using the DuoSet ELISA Development System for human CCL8/MCP-2 from R&D Systems (Cat. No. DY281).

Sample, reagent, and buffer preparation were done according to manufacturer manuals, and the assay was performed by following manual instructions exactly. Microwell plates were read by a SPECTRAMax 190 microplate reader (Molecular Devices, Sunnyvale, CA). Protein concentrations were determined from a standard curve generated from standards supplied with the kits. Protein concentrations of PECAM1, CXCL9 and CD44 in the plasma samples of heart transplant patients were also measured with the ELISA kits specified above.

### Immunohistochemistry

Immunohistochemical staining was performed on 4 µm sections obtained from formalin-fixed paraffin embedded tissues using mouse monoclonal anti-human antibodies directed against PECAM-1 (DAKO, Carpinteria, CA; Catalog # M823; dilution 1∶150). Heat induced antigen retrieval was performed with Ventana Benchmark Autostainer. The staining was optimized using appropriate positive and negative controls.

### Statistical analysis

T-tests and chi-square tests were used to compare continuous and categorical clinical variables in patient demographics using SAS 9.1.3 (SAS Institute Inc., Cary, NC). Protein concentration data from ELISAs were compared between AR and STA using the Mann-Whitney U test in R. P-values ≤0.05 were considered statistically significant. The enrichment of known protein biomarkers in differentially expressed genes was calculated using Fisher's exact test in R.

## Supporting Information

Dataset S1 Predicted diagnostics protein biomarkers on 22 diseases.
(1.39 MB XLS)

Figure S1 Histogram of overlapping genes in three transplant rejection microarray datasets after shuffling gene labels. We shuffled the gene labels in the three pediatric renal, adult renal and cardiac transplant rejection gene expression data sets, calculated differentially expressed AR genes in common. After repeating the processed 100,000 times, we plotted the distribution of the number of overlapping genes (blue histogram). The probability of getting 17 or more common genes by random is less than 1% and the probability of getting 24 or more common genes is less than 1×10-5 (red curve).
(0.95 MB PDF)

Figure S2 Shared pathway for AR across solid-organ transplantation. Among 45 genes that were upregulated in the AR compared with stable biopsy samples across transplanted organs, 23 of them were involved in a single pro-inflammatory pathway regulated by STAT-1. We tested 5 proteins (circled) from the pathway by ELISA, and three of them (red circle) were validated as cross-organ serum protein biomarkers for transplant rejection. The 18 untested AR proteins from the 45 are highlighted in the pathway, providing promising leads for further validation. Five of them (red star) were known to have detectable levels of protein expression in the normal serum or urine according to our human biofluid proteome database. Seven of them (blue star) were studied in knock-out mouse models, confirming their involvement in the immune system.
(0.59 MB PDF)

Figure S3 ROC curves predicting renal and cardiac AR using PECAM1+CXCL9. ROC curves showed three-fold cross-validation results on predicting renal (solid curve) and cardiac (dotted curve) transplant rejection (AR) from stable graft function using a combined panel of PECAM1 and CXCL9 proteins in serum (renal) and plasma (cardiac). The true positive rates were showed as mean ± standard error across 1,000 three-fold cross-validation. It showed an improvement over individual proteins on cardiac AR and no improvement on renal AR.
(0.89 MB PDF)

Figure S4 Serum PECAM1 protein was significantly upregulated in AR than BK virus infection, chronic allograft injury, and stable graft function after renal transplant. The protein concentrations of PECAM1 was statistically significantly higher in the serum samples of 18 patients with acute rejection (AR) than 10 patients with BK virus infection (BKV), 10 patients with chronic allograft injury (CAN) and 20 patients with stable graft function (STA) serum samples after renal transplantation.
(1.40 MB PDF)

Table S1 Patient demographics of AR versus STA allograft biopsies in pediatric renal transplant microarray study.
(0.05 MB DOC)

Table S2 Forty-five AR genes commonly upregulated in biopsy-based gene expression studies across solid-organ transplantation.
(0.09 MB DOC)

Table S3 Patient demographics of renal transplant in ELISA study.
(0.05 MB DOC)

Table S4 Patient demographics of cardiac transplant in ELISA study.
(0.05 MB DOC)
