# Supplementary material for: Differentially Expressed RNA from Public Microarray Data Identifies Serum Protein Biomarkers for Cross-Organ Transplant Rejection and Other Conditions
Source: PLoS Comput Biol. 2010 Sep 23;6(9):e1000940. doi: 10.1371/journal.pcbi.1000940 (PMC2944782; doi:10.1371/journal.pcbi.1000940)

Fig. S1: Histogram of overlapping genes in three transplant rejection microarray datasets after shuffling gene labels

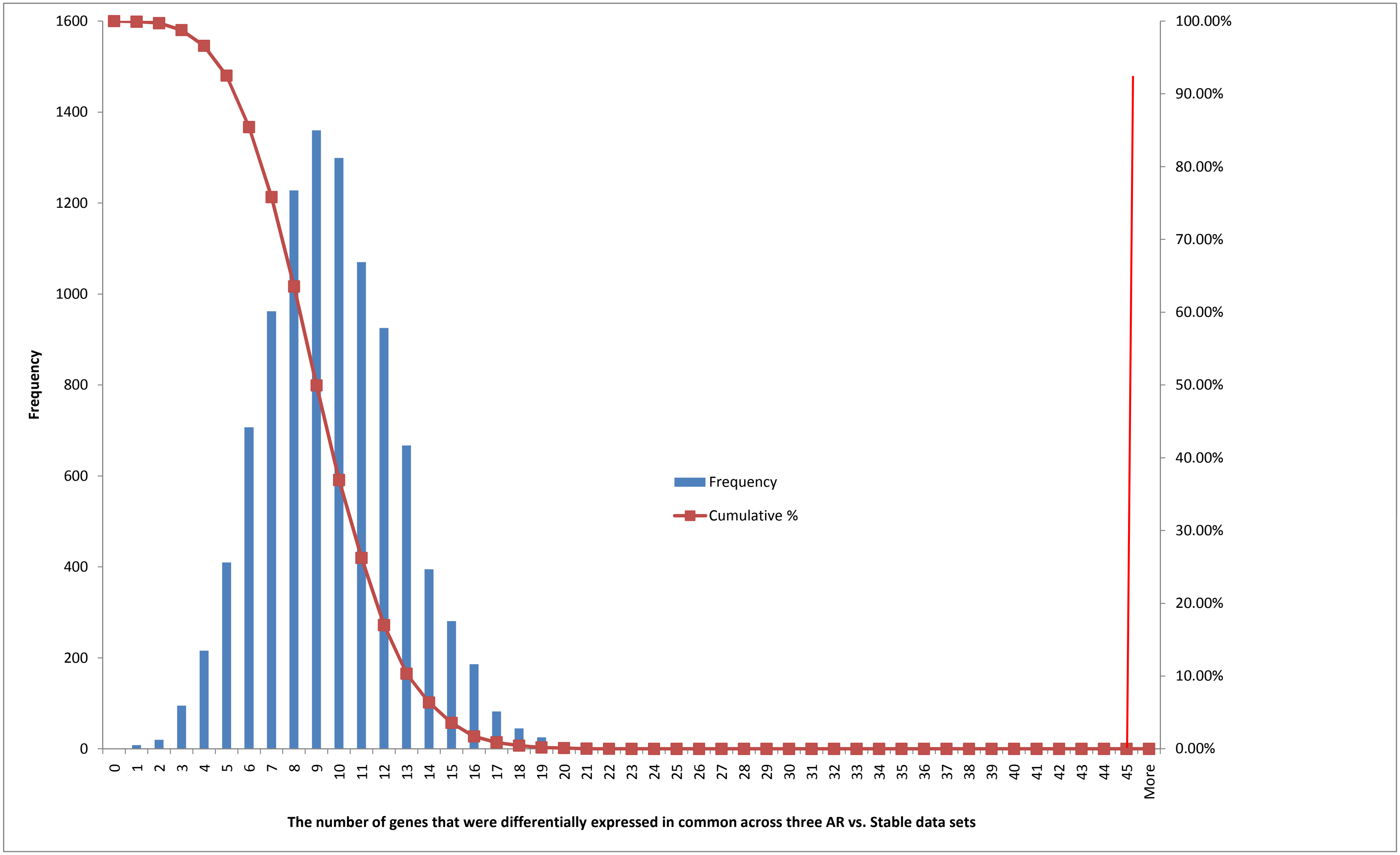

Supplement: Figure S1 — Histogram of overlapping genes in three transplant rejection microarray datasets after shuffling gene labels. We shuffled the gene labels in the three pediatric renal, adult renal and cardiac transplant rejection gene expression data sets, calculated differentially expressed AR genes in common. After repeating the processed 100,000 times, we plotted the distribution of the number of overlapping genes (blue histogram). The probability of getting 17 or more common genes by random is less than 1% and the probability of getting 24 or more common genes is less than 1×10^-5^ (red curve). (0.95 MB PDF) [file pcbi.1000940.s002.pdf]
