# Supplementary figures and images for: Differentially Expressed RNA from Public Microarray Data Identifies Serum Protein Biomarkers for Cross-Organ Transplant Rejection and Other Conditions
Source: PLoS Comput Biol. 2010 Sep 23;6(9):e1000940. doi: 10.1371/journal.pcbi.1000940 (PMC2944782; doi:10.1371/journal.pcbi.1000940)

**Fig. S3: ROC curves predicting renal and cardiac AR using PECAM1+CXCL9**

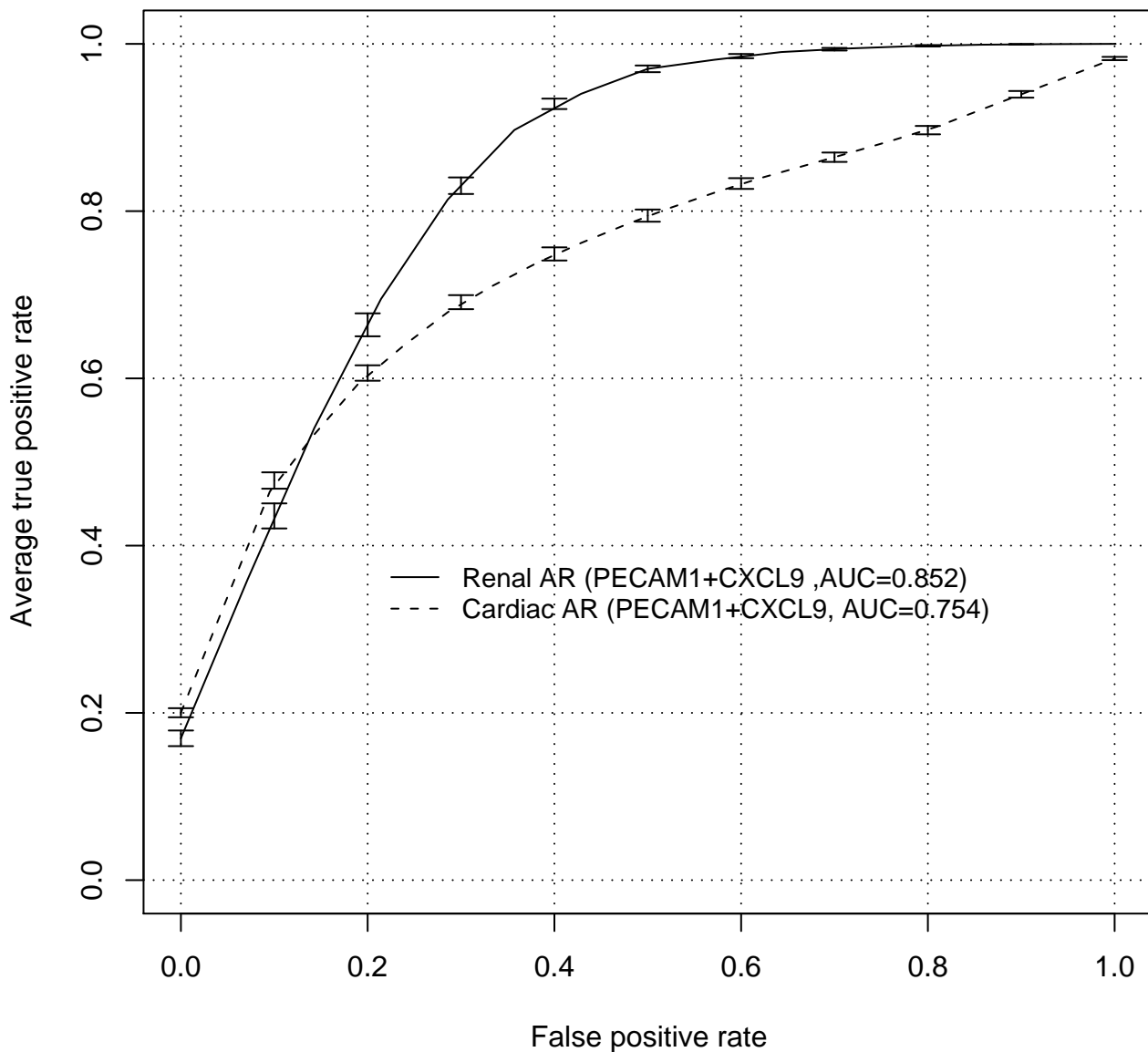

Supplement: Figure S3 — ROC curves predicting renal and cardiac AR using PECAM1+CXCL9. ROC curves showed three-fold cross-validation results on predicting renal (solid curve) and cardiac (dotted curve) transplant rejection (AR) from stable graft function using a combined panel of PECAM1 and CXCL9 proteins in serum (renal) and plasma (cardiac). The true positive rates were showed as mean ± standard error across 1,000 three-fold cross-validation. It showed an improvement over individual proteins on cardiac AR and no improvement on renal AR. (0.89 MB PDF) [file pcbi.1000940.s004.pdf]
