# Supplementary material for: Differentially Expressed RNA from Public Microarray Data Identifies Serum Protein Biomarkers for Cross-Organ Transplant Rejection and Other Conditions
Source: PLoS Comput Biol. 2010 Sep 23;6(9):e1000940. doi: 10.1371/journal.pcbi.1000940 (PMC2944782; doi:10.1371/journal.pcbi.1000940)

**Fig. S4: Serum PECAM1 protein was significantly upregulated in AR than BK virus infection, chronic allograft injury, and stable graft function after renal transplant**

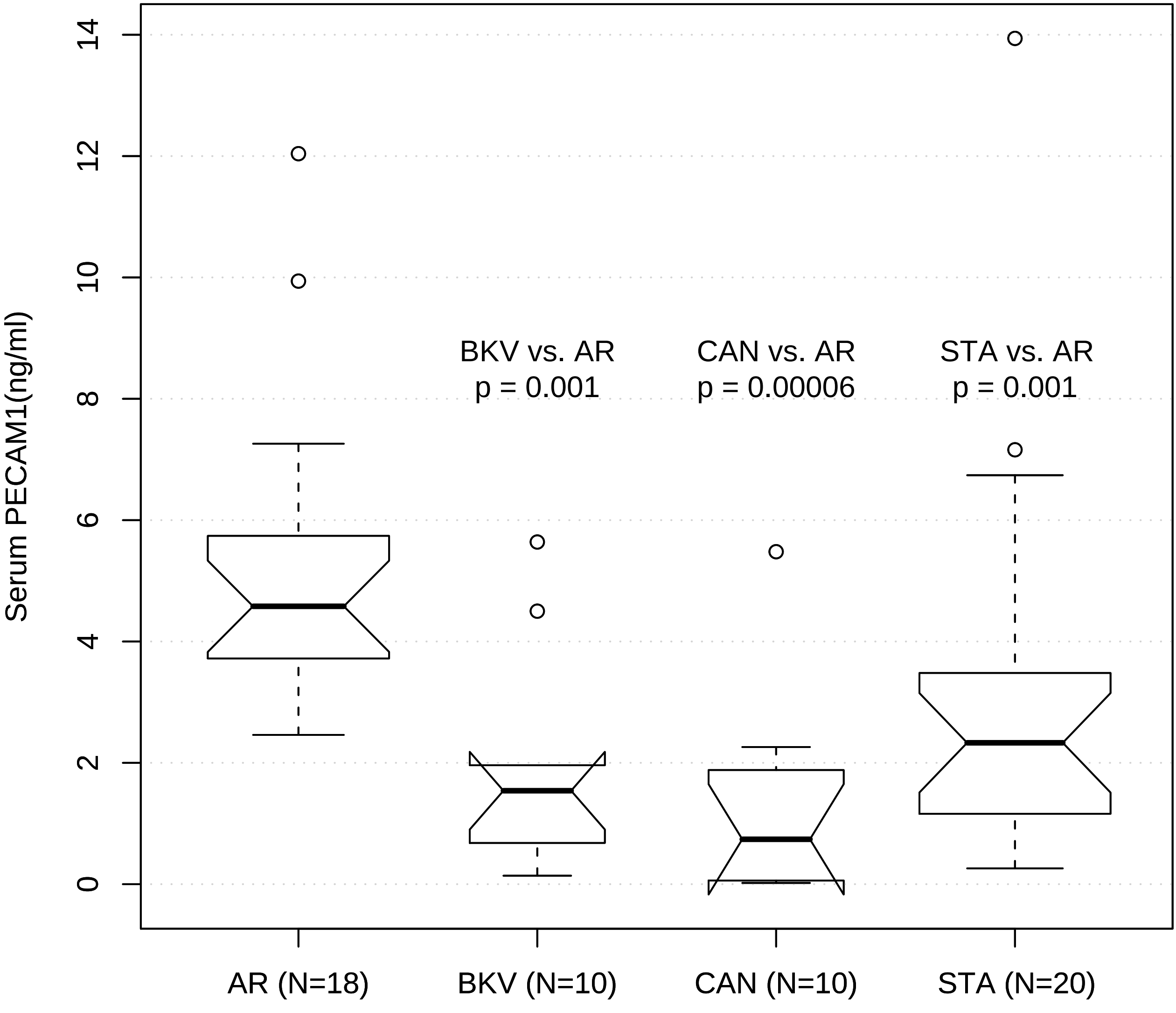

Supplement: Figure S4 — Serum PECAM1 protein was significantly upregulated in AR than BK virus infection, chronic allograft injury, and stable graft function after renal transplant. The protein concentrations of PECAM1 was statistically significantly higher in the serum samples of 18 patients with acute rejection (AR) than 10 patients with BK virus infection (BKV), 10 patients with chronic allograft injury (CAN) and 20 patients with stable graft function (STA) serum samples after renal transplantation. (1.40 MB PDF) [file pcbi.1000940.s005.pdf]
