# Supplementary material for: Differentially Expressed RNA from Public Microarray Data Identifies Serum Protein Biomarkers for Cross-Organ Transplant Rejection and Other Conditions
Source: PLoS Comput Biol. 2010 Sep 23;6(9):e1000940. doi: 10.1371/journal.pcbi.1000940 (PMC2944782; doi:10.1371/journal.pcbi.1000940)
Supplement: Table S1 — Patient demographics of AR versus STA allograft biopsies in pediatric renal transplant microarray study. (0.05 MB DOC) [file pcbi.1000940.s006.doc]

**Table S1**: Patient demographics of AR versus STA allograft biopsies in pediatric renal transplant microarray study

| **Clinical Characteristics** | **AR (n=18)** | **STA (n=18)** | **P value** |
| --- | --- | --- | --- |
| ***Recipients*** | | | |
| Gender, %females | 39% | 44% | 0.74 |
| Mean age (year) | 11.9 ± 6.1 | 11.7 ± 5.5 | 0.92 |
| Age range (year) | 1-21 | 1 - 19 |  |
| Immunosuppression, %SF^#^ | 61% | 33% | 0.1 |
| Sample collection time (month, post-transplant) | 17.4 ± 25.2 | 9.3 ± 6.1 | 0.2 |
| Sample collection time range (month, post-transplant) | 1 - 97 | 2 - 25 |  |
| Race(1,2,3,4,5)* | 73%,18%,0%,0%,25% | 35%,12%,12%,24%,17% | 0.18 |
| ESRD(1,2,3,4,5,6)** | 17%,8%,17%,41%,8%,8% | 17%,12%,18%,0%,6%,47% | 0.06 |
| ***Donors*** | | | |
| Source, %LRD | 50% | 59% | 0.6 |
| Gender, %females | 43% | 35% | 0.67 |
| Age (year) | 27.1 ± 13.6 | 29.7 ± 9.7 | 0.57 |
| Age range (year) | 4 - 44 | 15 - 55 |  |
| HLA match^$^ | 2.2 ± 1.8 | 1.2 ± 1.3 | 0.2 |

Values are mean ± SD (Standard Deviation)

AR: Acute Rejection; STA: stable; SF: Steroid-free drug treatment; ESRD: End stage renal disease; LRD: Living related donor

^#^Percentage of patients with steroid-free drug treatments (Method)

* Race: 1=Caucasian; 2= Hispanic; 3=Asian; 4=African American; 5=Other

**ESRD categories: 1=Glomerulonephritis; 2=Polycystic Kidney Disease; 3=Dysplasia; 4=Reflux Nephropathy; 5=Obstructive Uropathy; 6=Other

^$^The number of matched HLA pairs on A1, A2, B1, B2, DR2 between donor and recipient.
