# Supplementary material for: Differentially Expressed RNA from Public Microarray Data Identifies Serum Protein Biomarkers for Cross-Organ Transplant Rejection and Other Conditions
Source: PLoS Comput Biol. 2010 Sep 23;6(9):e1000940. doi: 10.1371/journal.pcbi.1000940 (PMC2944782; doi:10.1371/journal.pcbi.1000940)
Supplement: Table S2 — Forty-five AR genes commonly upregulated in biopsy-based gene expression studies across solid-organ transplantation. (0.09 MB DOC) [file pcbi.1000940.s007.doc]

**Table S2**: Forty-five AR genes commonly upregulated in biopsy-based gene expression studies across solid-organ transplantation

| **Gene** | **Pediatric Kidney AR** | | **Adult**  **kidney AR** | | **Heart AR** | | **Human Proteome Databases** | **Tested by ELISA** | **AR Biomarker** |
| --- | --- | --- | --- | --- | --- | --- | --- | --- | --- |
|  | **Fold** | **Q** | **Fold** | **q** | **Fold** | **q** |  |  |  |
| CXCL9 | 4.0 | 0 | 3.1 | 1×10^-3^ | 14.7 | 0 |  | Y | Y |
| CXCL11 | 4.9 | 0 | 1.7 | 0.02 | 6.3 | 5×10^-3^ |  | Y |  |
| CXCR4 | 8.7 | 0 | 1.5 | 0.02 | 2.4 | 9×10^-3^ |  |  |  |
| STAT-1 | 7.0 | 0 | 2.3 | 0 | 3.0 | 0 | Plasma | Y |  |
| CCL4 | 3.5 | 0 | 4.2 | 1×10^-3^ | 3.0 | 0.03 | Plasma | Y |  |
| C6orf32 | 4.4 | 0 | 4.2 | 3×10^-3^ | 1.8 | 5×10^-3^ |  |  |  |
| MARCKS | 6.5 | 0 | 1.4 | 0.04 | 2.3 | 0 | Urine, Plasma |  |  |
| IGSF6 | 4.3 | 0 | 2.3 | 0.02 | 3.2 | 0 |  |  |  |
| CD2 | 2.1 | 6×10^-3^ | 1.3 | 5×10^-4^ | 6.2 | 0 |  |  |  |
| TRPM1 | 6.2 | 7×10^-5^ | 1.1 | 0.04 | 1.7 | 0.03 | Plasma |  |  |
| IL10RA | 5.3 | 0 | 1.1 | 0.03 | 1.8 | 0.03 |  |  |  |
| RARRES3 | 2.7 | 0 | 1.4 | 3×10^-3^ | 4.0 | 0 |  |  |  |
| NR4A2 | 5.3 | 0 | 1.3 | 0.02 | 1.4 | 0.04 |  |  |  |
| PTPRC | 2.5 | 0 | 2.2 | 9×10^-4^ | 3.2 | 0 |  |  |  |
| LEF1 | 2.7 | 2×10^-4^ | 1.7 | 0.01 | 3.2 | 0 |  |  |  |
| TAP1 | 2.7 | 0 | 1.4 | 2×10^-3^ | 3.3 | 0 |  |  |  |
| CTSS | 3.5 | 0 | 1.6 | 0.01 | 2.3 | 5×10^-3^ | Plasma |  |  |
| ISG20 | 2.4 | 4×10^-4^ | 1.3 | 0.03 | 3.6 | 0 |  |  |  |
| CCL8 | 3.9 | 7×10^-5^ | 1.1 | 0.03 | 2.1 | 0.03 |  | Y |  |
| BASP1 | 2.8 | 7×10^-5^ | 2.0 | 0.01 | 2.0 | 0.04 | Urine, Plasma |  |  |
| SLC2A3 | 2.8 | 0 | 2.0 | 0.01 | 1.8 | 0 |  |  |  |
| LCP2 | 2.5 | 0 | 2.3 | 3×10^-3^ | 1.6 | 0.03 |  |  |  |
| HLA-DMA | 2.3 | 1×10^-3^ | 1.4 | 3×10^-3^ | 2.7 | 5×10^-3^ |  |  |  |
| BIRC5 | 3.1 | 0 | 1.1 | 0.02 | 2.2 | 0.02 |  | Y |  |
| HLA-DMB | 2.4 | 2×10^-4^ | 1.4 | 1×10^-3^ | 2.4 | 0 |  |  |  |
| CASP4 | 2.1 | 4×10^-4^ | 1.8 | 1×10^-3^ | 2.3 | 0 |  |  |  |
| SELL | 2.0 | 3×10^-3^ | 1.3 | 0.04 | 2.9 | 0 | Plasma | Y |  |
| HLA-F | 2.2 | 0 | 1.3 | 0.02 | 2.6 | 0 |  |  |  |
| CD44 | 3.5 | 0 | 1.1 | 0.03 | 1.5 | 0.02 | Urine, Plasma | Y | Y |
| HLA-DQB1 | 2.6 | 0 | 1.5 | 0.01 | 2.1 | 0.02 |  |  |  |
| PIK3CD | 2.1 | 0 | 1.1 | 0.03 | 2.7 | 0 |  |  |  |
| SH2D2A | 2.3 | 0 | 1.1 | 0.02 | 2.4 | 5×10^-3^ |  |  |  |
| CCNB2 | 2.4 | 5×10^-4^ | 1.2 | 2×10^-3^ | 2.1 | 5×10^-3^ | Plasma |  |  |
| HLA-DRA | 2.0 | 0.01 | 1.3 | 9×10^-4^ | 2.3 | 5×10^-3^ |  |  |  |
| B2M | 2.2 | 7×10^-5^ | 1.2 | 0.01 | 2.2 | 5×10^-3^ | Urine |  |  |
| DIAPH1 | 2.8 | 7×10^-3^ | 1.1 | 0.03 | 1.7 | 9×10^-3^ | Plasma |  |  |
| USP34 | 2.1 | 0 | 1.8 | 1×10^-3^ | 1.7 | 0.03 | Plasma |  |  |
| SCAND2 | 2.7 | 7×10^-4^ | 1.3 | 5×10^-4^ | 1.6 | 0.02 |  |  |  |
| RUNX1 | 2.2 | 0 | 1.2 | 3×10^-3^ | 2.1 | 0.03 |  |  |  |
| S100A4 | 2.6 | 7×10^-5^ | 1.1 | 0.02 | 1.6 | 0.03 | Urine | Y |  |
| PECAM1 | 2.4 | 0 | 1.3 | 9×10^-4^ | 1.5 | 0.02 | Urine, Plasma | Y | Y |
| MDK | 2.1 | 5×10^-4^ | 1.1 | 0.02 | 1.8 | 5×10^-3^ | Plasma |  |  |
| MELK | 2.0 | 4×10^-4^ | 1.2 | 3×10^-3^ | 1.7 | 0.02 |  |  |  |
| CDKN3 | 2.3 | 0 | 1.1 | 0.02 | 1.4 | 5×10^-3^ |  |  |  |
| CPD | 2.0 | 4×10^-3^ | 1.2 | 0.02 | 1.4 | 0.04 | Plasma |  |  |
