# Supplementary material for: Differentially Expressed RNA from Public Microarray Data Identifies Serum Protein Biomarkers for Cross-Organ Transplant Rejection and Other Conditions
Source: PLoS Comput Biol. 2010 Sep 23;6(9):e1000940. doi: 10.1371/journal.pcbi.1000940 (PMC2944782; doi:10.1371/journal.pcbi.1000940)
Supplement: Table S3 — Patient demographics of renal transplant in ELISA study. (0.05 MB DOC) [file pcbi.1000940.s008.doc]

| **Table S3**: Patient demographics of renal transplant in ELISA study   | **Clinical Characteristics** | **AR (n=19)** | **STA (n=20)** | **P value** | | --- | --- | --- | --- | | ***Recipients*** | | | | | Gender, %females | 53% | 65% | 0.43 | | Mean age (year) | 11.5 ± 6.5 | 13.6 ± 4.1 | 0.24 | | Age range (year) | 1.5 - 18.7 | 2.4 – 19.0 |  | | Immunosuppression, %SF^#^ | 32% | 50% | 0.24 | | Sample collection time (month, post-transplant) | 8.4 ± 6.7 | 6.2 ± 1.4 | 0.17 | | Sample collection time range (month, post-transplant) | 0.1 – 65.0 | 5.4 – 12.0 |  | | Race(1,2,3,4,5)* | 37%,0%,0%,47%,16% | 60%,0%,0%,25%,15% | 0.32 | | ESRD(1,2,3,4,5,6)** | 21%,0%,11%,5%,5%,58% | 11%,5%,5%,16%,5%,58% | 0.81 | | ***Donors*** | | | | | Source, %LRD | 32% | 55% | 0.14 | | Gender, %females | 37% | 45% | 0.6 | | Age (year) | 26.7 ± 9.4 | 30.7 ± 11.6 | 0.24 | | Age range (year) | 5 – 42 | 17 – 54 |  | | HLA match^$^ | 1.0 ± 1.3 | 1.9 ± 1.3 | 0.1 |   Values are mean ± SD (Standard Deviation)  AR: Acute Rejection; STA: stable; SF: Steroid-free drug treatment; ESRD: End stage renal disease  LRD: Living related donor  ^#^Percentage of patients with steroid-free drug treatments (Method)  * Race: 1=Caucasian; 2= Hispanic; 3=Asian; 4=African American; 5=Other  **ESRD categories: 1=Glomerulonephritis; 2=Polycystic Kidney Disease; 3=Dysplasia;  4=Reflux Nephropathy; 5=Obstructive Uropathy; 6=Other  ^$^The number of matched HLA pairs on A1, A2, B1, B2, DR2 between donor and recipient. |  |  |
| --- | --- | --- | --- | --- | --- | --- | --- | --- | --- | --- | --- | --- | --- | --- | --- | --- | --- | --- | --- | --- | --- | --- | --- | --- | --- | --- | --- | --- | --- | --- | --- | --- | --- | --- | --- | --- | --- | --- | --- | --- | --- | --- | --- | --- | --- | --- | --- | --- | --- | --- | --- | --- | --- | --- | --- | --- | --- | --- | --- | --- | --- | --- | --- | --- | --- | --- |
