# Supplementary material for: Differentially Expressed RNA from Public Microarray Data Identifies Serum Protein Biomarkers for Cross-Organ Transplant Rejection and Other Conditions
Source: PLoS Comput Biol. 2010 Sep 23;6(9):e1000940. doi: 10.1371/journal.pcbi.1000940 (PMC2944782; doi:10.1371/journal.pcbi.1000940)
Supplement: Table S4 — Patient demographics of cardiac transplant in ELISA study. (0.05 MB DOC) [file pcbi.1000940.s009.doc]

| **Table S4**: Patient demographics of cardiac transplant in ELISA study |  |  |
| --- | --- | --- |
| |  | **AR (n=32)** | **STA (n=31)** | **P value** |  | | | | --- | --- | --- | --- | --- | --- | --- | | ***Recipients*** | | | |  | | | | Gender, %females | 28% | 19% | 0.41 |  | | | | Mean age (year) | 40.6 ± 16.3 | 46.1 ± 19.3 | 0.22 |  | | | | Age range (year) | 11 - 66 | 10 - 69 |  |  | | | | Diastolic blood pressure | 78.3 ± 9.1 | 77.7 ± 17.8 | 0.87 |  | | | | Systolic blood Pressure | 124.8 ± 14.0 | 125.0 ± 26.7 | 0.98 |  | | | | Weight (kg) | 92.8 ± 21.6 | 83.1 ± 24.6 | 0.1 |  | | | | Sample collection time (month, post-transplant) | 10.0 ± 9.8 | 9.2 ± 5.4 | 0.71 |  | | | | Sample collection time range (month, post-transplant) | 0.4 - 41 | 0.8 - 18 |  |  | | | | Race (1,2,3,4,5)* | 69%,9%,6%,16%,0% | 74%,13%,3%,10%,0% | 0.82 |  | | | | ESD(1,2,3,4)** | 59%,6%,22%,13% | 71%,3%,23%,3% | 0.5 |  | | | | ***Donors*** | | | |  | | | | Gender, %females | 22% | 19% | 0.8 |  | | | | Age (year) | 31.0 ± 16.7 | 29.1 ± 18.8 | 0.56 |  | | | | Age range (year) | 14 - 55 | 9 - 60 |  |  | | | | * Captions are the same as that in supplementary table 1  ** ESD= End-Stage Disease: ESD categories: 1= Cardiomyopathy; 2= Congenital Heart disease;  3=Ischemic Cardiomyopathy; 4=Valvular Heart Diseases | | | | |  |  | |  | | | | | | | | | |
