# Supplementary material for: Differentially Expressed RNA from Public Microarray Data Identifies Serum Protein Biomarkers for Cross-Organ Transplant Rejection and Other Conditions
Source: PLoS Comput Biol. 2010 Sep 23;6(9):e1000940. doi: 10.1371/journal.pcbi.1000940 (PMC2944782; doi:10.1371/journal.pcbi.1000940)

Fig. S2: Shared pathway for AR across solid-organ tranplantation

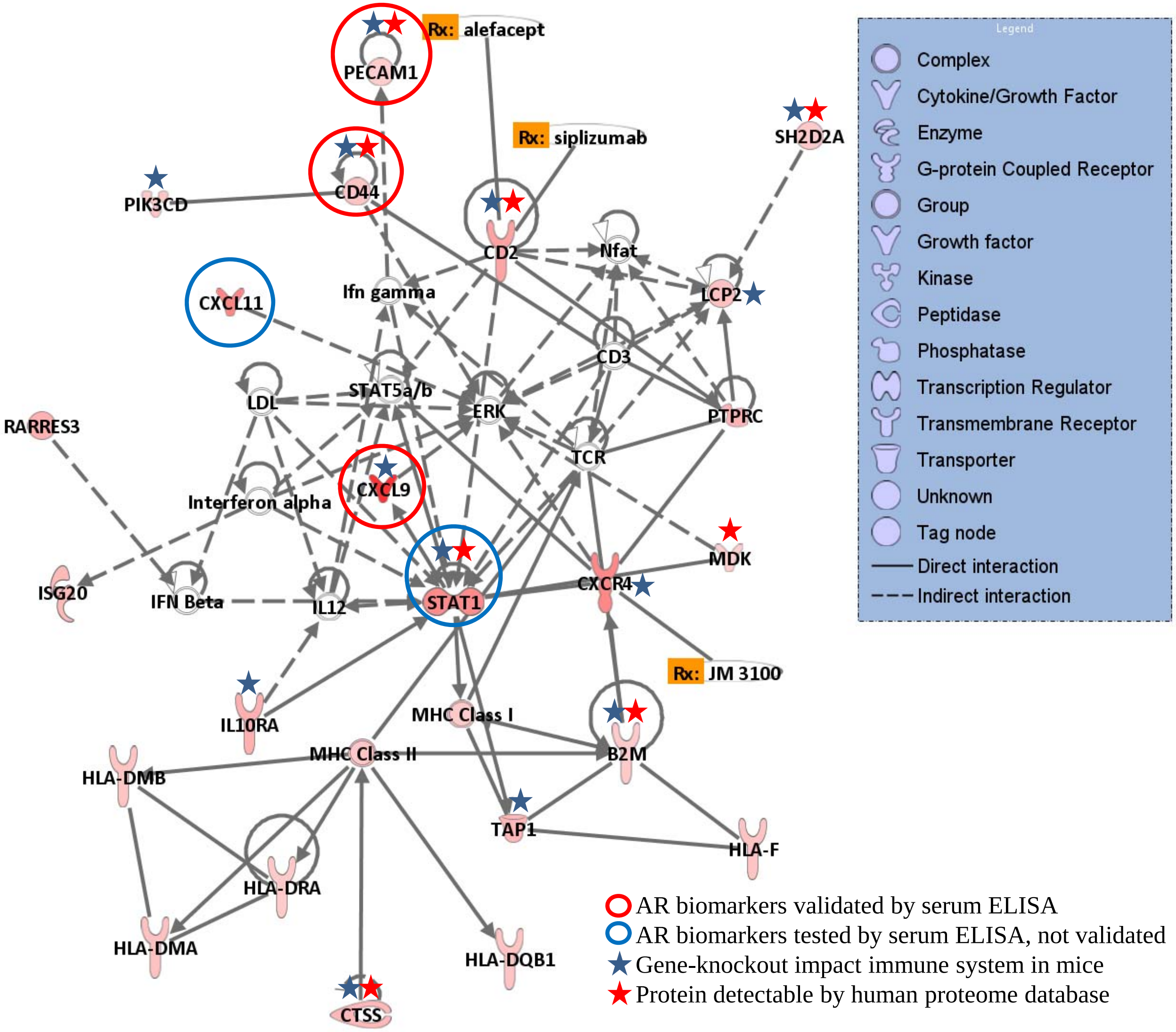

Supplement: Figure S2 — Shared pathway for AR across solid-organ transplantation. Among 45 genes that were upregulated in the AR compared with stable biopsy samples across transplanted organs, 23 of them were involved in a single pro-inflammatory pathway regulated by STAT-1. We tested 5 proteins (circled) from the pathway by ELISA, and three of them (red circle) were validated as cross-organ serum protein biomarkers for transplant rejection. The 18 untested AR proteins from the 45 are highlighted in the pathway, providing promising leads for further validation. Five of them (red star) were known to have detectable levels of protein expression in the normal serum or urine according to our human biofluid proteome database. Seven of them (blue star) were studied in knock-out mouse models, confirming their involvement in the immune system. (0.59 MB PDF) [file pcbi.1000940.s003.pdf]
